# The whole is faster than its parts: evidence for temporally independent attention to distinct spatial locations

**DOI:** 10.3758/s13414-015-1023-1

**Published:** 2015-11-24

**Authors:** Andrew Clement, Nestor Matthews

**Affiliations:** Department of Psychology, University of Notre Dame, Notre Dame, IN 46556 USA; Department of Psychology, Denison University, Granville, OH 43023 USA

**Keywords:** Visual attention, Episodic sampling, RSVP, Left visual field advantage, Temporal Resolution, When Pathway, Entrainment, Masking, Hemifield Asymmetry

## Abstract

Behavioral and electrophysiological evidence suggests that visual attention operates in parallel at distinct spatial locations and samples the environment in periodic episodes. This combination of spatial and temporal characteristics raises the question of whether attention samples locations in a phase-locked or temporally independent manner. If attentional sampling rates were phase locked, attention would be limited by a global sampling rate. However, if attentional sampling rates were temporally independent, they could operate additively to sample higher rates of information. We tested these predictions by requiring participants to identify targets in 2 or 4 rapid serial visual presentation (RSVP) streams, synchronized or asynchronized to manipulate the rate of new information globally (across streams). Identification accuracy exhibited little or no change when the global rate of new information doubled from 7.5 to 15 Hz (Experiment 1) or quadrupled to 30 Hz (Experiment 2). This relatively stable identification accuracy occurred even though participants reliably discriminated 7.5 Hz synchronous displays from displays globally asynchronized at 15 and 30 Hz (Metamer Control Experiment). Identification accuracy in the left visual field also significantly exceeded that in the right visual field. Overall, our results are consistent with temporally independent attention across distinct spatial locations and support previous reports of a right parietal “when” pathway specialized for temporal attention.

Subjective experience suggests that we view the world continuously, with few lapses in attentional input. However, research on attention’s temporal properties suggests otherwise. As early as 1910, psychologists posited that attention is deployed in episodic intervals (Tichener, 1910). Although support for this claim has been intermittent, recent psychophysical studies provide evidence for episodic sampling in visual attention. Much of this evidence comes from experiments that embed two targets in rapid serial visual presentation (RSVP) displays. While participants often incorrectly report the temporal order of targets separated by ∼100 ms, their performance improves at longer target asynchronies (Akyürek, Riddell, Toffanin, & Hommel, 2007; Chun, 1997; Chun & Potter, 1995; Isaak, Shapiro, & Martin, 1999; Olivers, Hilkenmeier, & Scharlau, 2011; Reeves & Sperling, 1986; Spalek, Falcon, & Di Lollo, 2006; Spalek, Lagroix, Yanko, & Di Lollo, 2012). The duration of this effect can vary, depending on the number of targets and the presence of intervening distractors (Akyürek, Toffanin, & Hommel, 2008; Kawahara, Kumada, & Di Lollo, 2006; Olivers, Van der Stigchel, & Hulleman, 2007; Visser, 2015). Nonetheless, based on recent computational work, this effect suggests that stimuli in close temporal succession become integrated into brief attentional episodes (Akyürek et al., 2012; Wyble, Bowman, & Nieuwenstein, 2009; Wyble, Potter, Bowman, & Nieuwenstein, 2011). Notably, these episodes are consistent with other psychophysical evidence, which reveals brief intervals of attentional sampling in many visual attention tasks (Dugué & Vanrullen, 2014; Landau & Fries, 2012; VanRullen, Carlson, & Cavanagh, 2007). Based on such findings, many researchers have concluded that visual attention operates in an episodic fashion (Akyürek et al., 2012; VanRullen & Dubois, 2011; VanRullen & Koch, 2003; Wyble et al., 2011).

The previous evidence suggests that attention is deployed in brief episodes. Electrophysiological evidence also suggests that these episodes occur periodically. Oscillations in neural activity have long been assumed to play a role in attention (Niebur, Koch, & Rosin, 1993; Womelsdorf & Fries, 2007), and several recent studies reveal oscillations in scalp recordings during visual attention tasks (Busch, Dubois, & VanRullen, 2009; Capotosto et al., 2015; Chakravarthi & VanRullen, 2012; Gray, Frey, Wilson, & Foxe, 2015; Hanslmayr et al., 2007; Mathewson, Gratton, Fabiani, Beck, & Ro, 2009; Spaak, De Lange, & Jensen, 2014 ). For example, visual detection and discrimination have been shown to fluctuate with prestimulus oscillatory phase, with waveform peaks predicting increased performance and waveform troughs predicting decreased performance (Busch et al., 2009; Hanslmayr, Volberg, Wimber, Dalal, & Greenlee, 2013; Mathewson et al., 2009). Other studies have found modulation of waveform power during similar attentional tasks (Hanslmayr et al., 2007; Marshall, O’Shea, Jensen, & Bergmann 2015). Moreover, neural oscillations have been found to specifically predict visual detection at attended locations (Busch & VanRullen, 2010). These findings corroborate several behavioral studies, which suggest that attention samples one or more locations at consistent rates of 7 to 10 Hz (Dugué & Vanrullen, 2014; Landau & Fries, 2012; VanRullen et al., 2007). Together, these results support the notion that visual attention is deployed in periodic episodes.

To account for these findings, several models of visual attention have been proposed. Given the consistent sampling rates observed in previous behavioral studies, some of these models assume a single focus of attention. For example, the “blinking spotlight” model posits that a single attentional resource samples multiple locations in successive temporal intervals (VanRullen et al., 2007; VanRullen & Dubois, 2011). Unlike competing models, this model assumes that attentional resources cannot be divided among multiple locations at once (a “multiple spotlights” account; Bay & Wyble, 2014; McMains & Somers, 2004; Gray et al., 2014; Spaak et al., 2014). This distinction calls to mind the long-standing debate between unitary (serial) and divided (parallel) attention. Although this debate is ongoing (Dubois, Hamker, & VanRullen, 2009; Jans, Peters, & De Weerd, 2010; Jefferies, Enns, & Di Lollo, 2014), a growing body of evidence suggests that attention may be deployed in parallel. For example, using partial report and RSVP tasks, researchers have found simultaneous attentional enhancement at multiple cued locations (Awh & Pashler, 2000; McMains & Somers, 2004, 2005). By employing rapid presentation rates and visual masking, these studies prevented serial switching among locations, thus supporting the presence of multiple attentional foci. Interestingly, attentional enhancement in these studies was greatest when cued locations were divided across the left and right visual hemifields (LVF and RVF, respectively; but see Bay & Wyble, 2014). Subsequent studies have corroborated these findings. For example, using an attentional tracking task, Alvarez and Cavanagh (2005) found that people could track twice as many targets when they were divided across lateral hemifields. Similarly, multistream RSVP studies have shown increased attentional performance when targets appear in opposite hemifields (Scalf et al., 2007). Together, these bilateral advantages suggest that parallel resources control attention in the LVF and RVF (Alvarez & Cavanagh, 2005; Chakravarthi & Cavanagh, 2009; Reardon, Kelly, & Matthews, 2009; Alvarez, Gill, & Cavanagh, 2012).

If attentional resources operate independently in the LVF and RVF, there are several implications for episodic models of attention. Most importantly, the assumption of a single attentional focus must be revised. To address this problem, VanRullen et al. (2007) suggested a hybrid model in which attention is deployed simultaneously across hemifields while periodically sampling locations within each hemifield. However, such a model raises the question of whether attention samples multiple locations in a phase-locked or temporally independent manner. To appreciate this issue, consider a display in which stimuli must be simultaneously monitored in the LVF and RVF. Although attention may be deployed in parallel to both hemifields, the rate of attentional sampling could be set either across hemifields (a phase-locked system) or within hemifields (a temporally independent system). In a phase-locked system, LVF and RVF information would be sampled at the same time, regardless of whether stimuli appear simultaneously. However, in a temporally independent system, LVF and RVF information would be sampled individually, allowing attention to sample spatially distinct stimuli at separate time points. Note that at sufficiently large time scales, these two systems would sample the same amount of information per unit time; their distinct temporal characteristics would become discernable only when analyzing sufficiently small time scales.

Based on existing evidence, there is reason to believe that attention is deployed in a temporally independent fashion. Much of this evidence comes from lateral asymmetries in visual attention. For example, in multi-stream RSVP tasks, there is often improved attentional performance when targets appear in the LVF (Holländer, Corballis, & Hamm, 2005; Scalf, Banich, Kramer, Narechania, & Simon, 2007; Śmigasiewicz et al., 2010; Verleger et al., 2009). Similar LVF advantages have been shown for simultaneity, motion direction, and temporal order judgments, with greater precision occurring for LVF targets (Kelly & Matthews, 2011; Matthews, Vawter, & Kelly, 2012; Bosworth, Petrich, & Dobkins, 2012; Matthews & Welch, 2015). These findings, along with impaired temporal judgments in right parietal lobe patients, have led some researchers to speculate about a right parietal “when” pathway specialized for temporal attention (Battelli, Pascual-Leone, & Cavanagh, 2007; Battelli, Walsh, Pascual-Leone, & Cavanagh, 2008; Davis, Christie, & Rorden, 2009). If such a pathway exists, attentional sampling rates in the LVF could be set independently of those in the RVF. Recent event-related potential (ERP) data provide preliminary support for this claim, indicating that N2pc (parietal contralateral) components peak earlier for LVF targets in dual-stream RSVP tasks (Verleger, Dittmer, & Śmigasiewicz, 2013; Verleger, Smigasiewicz, & Moller, 2011). Given that N2pc components are reliable markers of attentional selection (Hopf et al., 2000; Anderson, Ester, Klee, Vogel, & Awh, 2014), this finding demonstrates that LVF advantages are linked to changes in temporal attention. A recent study in our own laboratory supports this claim, showing that LVF targets are perceived earlier than RVF targets in temporal order judgments (Matthews, Welch, Festa, & Clement, 2013).

Although the previous findings support the presence of independent sampling rates, no study has explicitly tested attention’s temporal independence. To explore this possibility, we sought to measure attention to spatially distinct, rapidly presented stimuli. If attention samples locations in a phase-locked manner, attention should be limited by a global sampling rate. However, if attention samples locations in a temporally independent manner, attentional resources could operate additively to sample higher rates of new information. For example, if temporally independent resources become entrained to separate 10 Hz rhythms (as in Spaak et al., 2014), they could attend to global information rates that exceed this range. Given two rhythms in counterphase, these resources could sample global information rates of up to 20 Hz – approximately twice the sampling rates observed in some behavioral studies (VanRullen et al., 2007; Dugué & Vanrullen, 2014; Landau & Fries, 2012). To illustrate this principle, consider Wever and Bray’s (1937) volley theory, which explains how limited neural firing rates register high auditory frequencies. According to the theory, distinct neural ensembles synchronize their activity at various phases of an auditory stimulus. This neural-ensemble volleying yields collective firing rates well above those of individual neurons, enabling the perception of higher auditory frequencies (Wever, 1949; see Fig. 1). Note that the volley theory is unlikely to rely on a temporally independent mechanism, as synchronizing to different phases of a stimulus implies a certain level of temporal dependence. However, like volleying neural ensembles, temporally independent resources could additively improve an organism’s temporal precision.

**Fig. 1:**
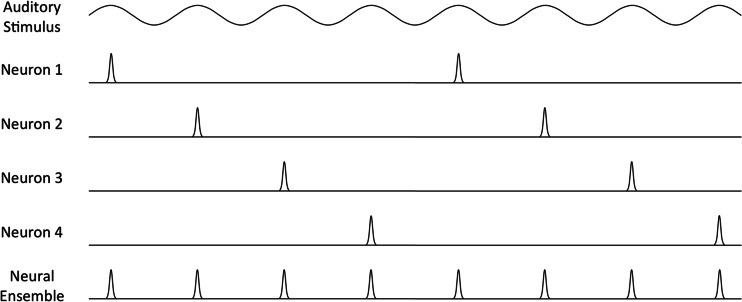
Illustration of Wever and Bray’s (1937) volley theory. Individual neurons become entrained to different phases of an auditory stimulus, producing offsets in neural firing rates. This alternating activity allows the entire ensemble to volley at four times the rate of the individual neurons, effectively matching the stimulus frequency

In the present study, we investigated whether parallel attentional resources operate in a phase-locked or temporally independent fashion. To address this question, we employed multistream RSVP tasks similar to those used by Verleger et al. (2011) and Scalf et al. (2007). On each trial, participants were asked to identify two sequentially presented targets (T1 and T2) embedded in two or four stimulus streams. In each stream, new information occurred at a rate of 7.5 Hz. This information rate was selected based on previous RSVP work, which revealed accurate attentional sampling at similar information rates (Holländer et al., 2005; Matthews et al., 2013; Matthews & Welch, 2015; Scalf et al., 2007; Śmigasiewicz et al., 2010; Verleger et al., 2009) as well as LVF behavioral advantages (Matthews et al., 2013) and related changes in N2pc attentional markers (Verleger et al., 2013; Verleger et al. 2011). In a critical manipulation, visual information in the streams was presented either synchronously (in phase) or asynchronously (out of phase). By asynchronizing information across streams, we increased the rate at which new information occurred globally while maintaining 7.5 Hz presentation rates locally. This procedure allowed us to determine whether target identification was impaired at global information rates greater than 7.5 Hz. If this were the case, our results would point to a global limit on attentional sampling, suggesting phase locking across distinct spatial locations. However, if target identification rates were similar for the synchronized and asynchronized conditions, this would suggest temporal independence across distinct spatial locations.

To summarize our findings, participants performed comparably in the synchronized and asynchronized conditions, matching the predictions that would follow from temporal independence. This effect occurred regardless of whether we doubled or quadrupled the global rate of new information. We also observed a significant LVF advantage in T2|T1 identification. Overall, our results point to temporally independent attention across distinct spatial locations, and support previous reports of a right parietal “when” pathway specialized for temporal attention.

## General method

### Participants

Denison University’s Human Subject Committee approved all experiments in this study, which we conducted with the understanding and written consent of each participant. The participants – Denison University undergraduates who reported normal or corrected vision – possessed no prior knowledge about the hypotheses and received either course credit or financial compensation for their time. Across experiments, the sample size (see Table 1) fluctuated with participant availability during the academic semesters and summer. Some participants completed more than one experiment.

**Table 1:** Sample size for each experiment

Experiment 1	Experiment 2	Metamer Control
*N* = 19	*N* = 26	*N* = 16

### Apparatus

Dell OptiPlex 780 desktop computers, each with a Microsoft Windows 7 Enterprise operating system, controlled the software. SuperLab 4.5 presentation software (Cedrus) controlled 17-in. (43.18-cm) flat-screen Dell 2009W displays, each with a 60-Hz vertical refresh rate and 1680 × 1050 spatial resolution. Although head position was not stabilized, participants typically viewed the monitor from a distance of ~57 cm.

## Experiment 1

### Stimuli

**Fig. 2:**
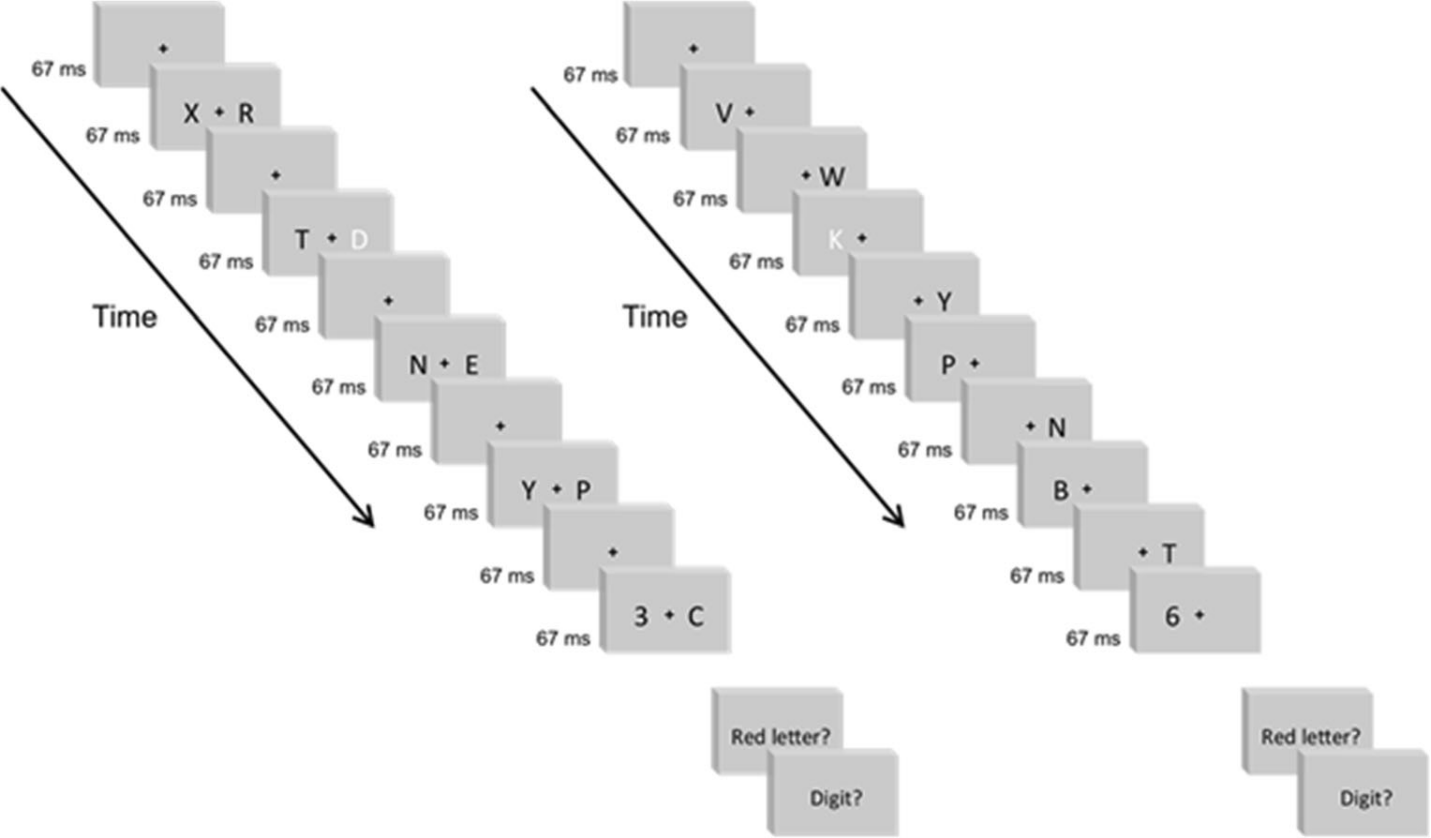
Sample frames from the dual-stream RSVP sequences in Experiment 1. Each RSVP sequence contained 20 bilaterally presented stimulus pairs comprising black letter distractors and two targets: T1 (a red letter – shown here in white) and T2 (a black digit). On synchronized trials (left panel), stimulus pairs flashed on odd-numbered frames. Even-numbered frames contained only the fixation cross. On asynchronized trials (right panel), stimuli alternated between the left and right hemifields on successive frames. In each condition, visual *transients* occurred globally at 15 Hz (every 67 ms) and *new* information occurred at 7.5 Hz (every 133 ms) within each hemifield. The rate at which *new* retinal information occurred globally distinguished the conditions: 7.5 Hz versus 15 Hz, respectively, on synchronized and asynchronized trials

**Fig. 3:**
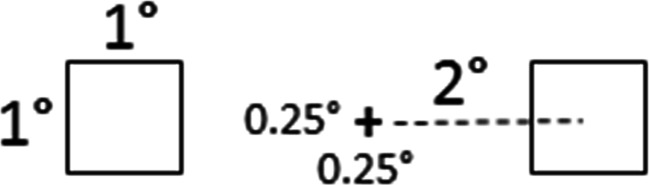
Size and spacing for the dual-stream RSVP displays in Experiment 1

The stimulus on each trial was a dual-stream RSVP sequence (see Fig. 2). Each sequence comprised forty 15-Hz frames (67 ms/frame; 2.667 s total), containing a black fixation cross (0.25° × 0.25°) centered in a white surround. Two degrees separated (center-to-center, horizontally) the fixation cross from each laterally flanking stimulus. The flanking stimuli were either Arabic numbers or capitalized English letters (Calibri font, stroke-width 0.02°) extending 1.0° vertically and a maximal 1.0° horizontally. (See Fig. 3 for stimulus size and spacing.) Each RSVP sequence contained two targets (T1 and T2) and 38 distractors. T1 was a red letter randomly selected on each trial from the set D, F, G, J, K, and L. T2 was a black digit randomly selected on each trial from the set 1, 2, 3, 4, 5, and 6. The distractors comprised all other letters, randomly sequenced, and always presented in black. On each trial, T1 and T2 each occurred only once, randomly positioned in either the LVF or RVF regardless of the other target’s hemifield location. This generated ipsilateral (LL or RR) and contralateral (RL or LR) target configurations equally often across trials.

In each dual-stream RSVP sequence, the left and right streams flashed either synchronously (see Fig. 2, left panel) or asynchronously (see Fig. 2, right panel). On synchronized trials, T2 lagged 400 ms (onset to onset; six frames at 66.67 ms/frame) behind T1, as two synchronized distractor pairs followed T1 and preceded T2. Because T2 was the third stimulus pair presented after T1, T2 would be said to occur at “Lag 3” in the RSVP literature. The same lag also pertained to asynchronized trials with ipsilateral targets (LL or RR). However, asynchronized trials with contralateral targets (RL or LR) necessarily required a slightly different temporal separation, given the left–right alternation across frames. Specifically, on asynchronized trials with contralateral targets, T2 lagged 333 ms (onset to onset; five frames at 66.67 ms/frame) behind T1, as two asynchronized distractor “pairs” followed T1 and preceded T2. In all cases, T1 flashed randomly between 1.267 and 1.733 s (frames 19 and 26) into the 2.667 s (40 frame) sequence. The supplementary information contains sample RSVP movies from all experiments conducted in this study.

### Task

After each dual-stream RSVP sequence, each participant first attempted to identify the red letter (T1), then the black digit (T2). Immediate error feedback followed each response. Participants responded on a standard computer keyboard with no restrictions on which finger or hand to use.

### Procedure

At the start of each trial block, written instructions on the monitor informed participants that a red letter (T1) would precede a black digit (T2) in a sequence of black-letter distractors. The written instructions also informed participants to maintain fixation on the central cross, as the targets would appear to the left and right equally often and randomly. Each participant then completed practice trials to become familiar with the RSVP stimuli and task. Subsequently, each participant completed five 80-trial blocks (400 trials for analysis). Each block comprised 10 trials in each of eight experimental conditions. These comprised two levels of the Timing variable (synchronized vs, asynchronized) crossed with two levels of the Side-Change variable (ipsilateral targets vs. contralateral targets) and two levels of the T2-Side variable (left vs. right). The computer randomized all conditions anew within each 80-trial block. In total, each participant completed 50 trials for analysis in each of the eight experimental conditions.

### Data analysis

**Fig. 4:**
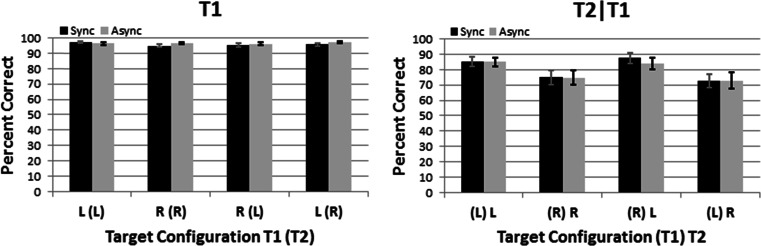
Data from the dual-stream RSVP displays in Experiment 1. Black and gray columns signify the synchronized and asynchronized Timing conditions, respectively. Ipsilateral targets (LL, RR) and contralateral targets (RL, LR) appear, respectively, on the left and right halves of each panel. Left and right panelsindicate the percentage correct for T1-identification and T2-identification given a correct T1 response (T2|T1), respectively. Error bars reflect +1 *SEM* (*N* = 19)

All statistics in this study reflect our completely within-subjects research designs. In Experiment 1, the independent variables were Timing (synchronized vs. asynchronized), Side-Change (ipsilateral targets vs. contralateral targets), and T2-Side (left vs. right). The dependent variables were the percentage of correct T1 identifications and, separately, T2 identifications given a correct T1 response.^1^ We used Bonferroni corrections to reduce cumulative Type I error across 15 planned (a priori) statistical comparisons in our 2 × 2 × 2 (Timing x Side-Change x T2-Side) experimental design. These 15 comparisons include three main effects, three two-way interactions, the three-way interaction, four Synchronized-vs.-Asynchronized *t* tests (one per target configuration in Fig. 4), and four Left-vs.-Right *t* tests (one per combination of Side-Change and T2-Side). Partial eta-squared (_p_η^*2*^ = SS_effect_ / [SS_effect_ + SS_error_]) indicates the effect size. The supplementary information contains the raw data from all experiments and all statistical analyses. For brevity in the text, we report the statistical analyses most directly relevant to our research questions.

## Results

Figure 4 shows the main finding from Experiment 1: virtually identical performance on synchronized (black bars) and asynchronized (gray bars) trials. This performance similarity occurred for T1-identification (left panel) and T2|T1-identification (right panel) alike. Stated differently, T1 and T2|T1-identification remained unaffected when we doubled the rate at which *new* retinal information occurred globally from 7.5 (synchronized condition) to 15 Hz (asynchonized condition). This finding argues against the possibility that global phase locking constrains visual attention’s temporal resolution. Instead, the performance similarities in our synchronized and asynchronized conditions can be explained by distinct LVF and RVF neural resources that are *temporally independent* from each other.

ANOVAs from Experiment 1 revealed only one significant effect. Specifically, on T2|T1-identification, LVF performance (85.4% correct) significantly exceeded RVF performance (73.5% correct), *F*(1, 18) = 15.2, *p* = .015, _p_η^2^ = 0.46. This LVF advantage matches prior reports that implicate the right parietal lobe’s unique role in temporal attention. Such reports include clinical data from split brain (Forster, Corballis, & Corballis, 2000) and right parietal lobe patients (Battelli et al., 2001; Battelli, Cavanagh, Martini, & Barton, 2003; Rorden, Mattingley, Karnath, & Driver, 1997), as well as physiological manipulations involving transcranial magnetic stimulation (TMS; Müri et al., 2002; Woo, Kim, & Lee, 2009). Again, these findings have generated speculation about a “when” pathway (Battelli et al., 2007; Battelli et al., 2008; Davis et al., 2009) that is distinct from the “what” (ventral) and “where” (dorsal) pathways (Mishkin & Ungerleider, 1982). Additional support for such a pathway comes from electophysiological (Verleger et al., 2013; Verleger et al., 2010, Verleger et al., 2011; Verleger et al. 2009) and psychophysical experiments that challenged attention’s temporal limits and revealed LVF advantages (Asanowicz, Smigasiewicz, & Verleger 2013; Bosworth et al., 2012; Holländer et al., 2005; Kelly & Matthews, 2011; Matthews et al., 2012; Matthews et al., 2013; Matthews & Welch, 2015; Scalf et al., 2007; Śmigasiewicz et al., 2010; but see Goodbourn & Holcombe, 2015). In any case, the LVF advantage observed in Experiment 1 argues against phase locking and supports temporally independent attentional resources for the LVF and RVF. Experiment 2 further explored temporal independence as a potential explanation for Experiment 1’s main finding that attention can operate at 15 Hz globally.

## Experiment 2

Experiment 1 revealed unimpaired RSVP target-identification accuracy when the global rate of new information was doubled from 7.5 to 15 Hz. On one hand, this finding could reflect temporal independence between neural resources dedicated to the left and right hemifields. Alternatively, this finding could reflect temporal independence across sufficiently distinct spatial locations, regardless of whether those locations occur in different hemifields. To distinguish between these possibilities, in Experiment 2 we presented multistream RSVP sequences similar to those used by Scalf et al. (2007). Each sequence contained four streams (one stream per visual quadrant) that flashed either synchronously or asynchronously. On synchronized trials, new information occurred at 7.5 Hz across three spatial scales: quadrant, hemifield, and globally. On asynchronized trials, new information occurred at 7.5 Hz per quadrant, 15 Hz per hemifield, and 30 Hz globally. A “temporally independent hemifields” hypothesis would predict significantly worse performance on asynchronized trials than on synchronized trials because a 15 Hz hemifield-presentation rate would exceed the presumed 7.5 Hz sampling rate per hemifield. Alternatively, a “temporally independent subhemifields” hypothesis would predict comparable performance across asynchronized and synchronized trials, as temporally independent 7.5 Hz sampling per quadrant could produce 15 Hz sampling per hemifield. In short, Experiment 2’s synchronized and asynchronized four-stream displays allowed us to probe temporal independence at hemifield versus subhemifield spatial scales.

## Method

### Stimuli

**Fig. 5:**
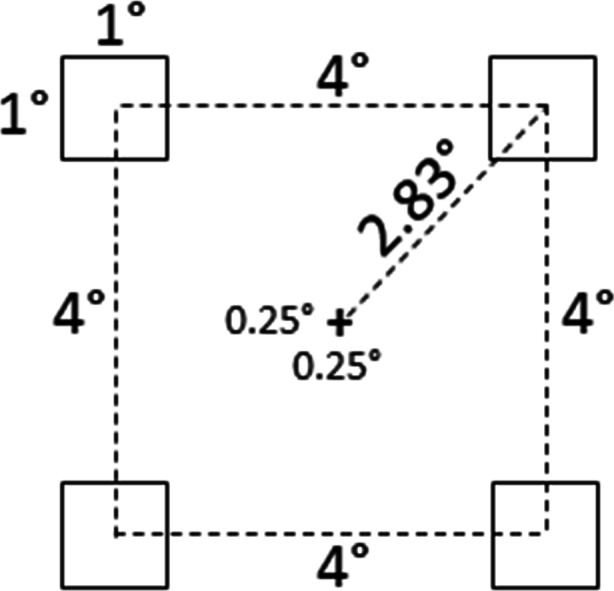
Size and spacing for the four-stream RSVP displays in Experiment 2

Experiment 2 comprised synchronized and asynchronized RSVP stimuli that, in several ways, differed from those of Experiment 1. First, the RSVP stimuli in Experiment 2 streamed across four quadrants. (See Fig. 5 for stimulus size and spacing.) Second, relative to Experiment 1, the frame rate in Experiment 2 doubled so that each RSVP sequence now comprised eighty 30 Hz frames (33.33 ms/frame; 2.667 s total). Synchronized RSVP sequences comprised sets of four stimuli (one stimulus per quadrant) presented *simultaneously* every fourth frame. The three temporally intervening “blank” frames contained only the fixation cross. Asynchronized RSVP sequences comprised stimuli presented *successively* across frames in different quadrants. We block randomized the quadrant sequence anew on each asynchronized trial such that, within any four consecutive frames, stimulation occurred in each quadrant once before occurring in any quadrant twice. The first quadrant was chosen randomly, and the second quadrant was diagonal to the first. The third and fourth quadrants were those above or below the first and second quadrants, respectively. The remaining frames on each trial adhered to this initial four-frame sequence. On all asynchronized trials, LVF and RVF quadrants always alternated across consecutive frames. Consequently, each asynchronized RSVP sequence generated *new* stimulus information at 7.5 Hz per quadrant, 15 Hz per lateral hemifield, and 30 Hz globally.^2^ Dissimilarly, synchronized RSVP sequences generated *new* stimulus information at 7.5 Hz at each spatial scale (i.e., quadrant, hemifield, and globally).

Regarding the targets, T1 and T2 occurred equally often in the upper and lower quadrants across trials but never occurred within the same quadrant on a given trial. Thus, on trials with ipsilateral targets (LL or RR), if T1 appeared in the upper left (right) quadrant, T2 would always appear in the lower left (right) quadrant. However, on trials with contralateral targets (RL or LR), if T1 appeared in the upper left (right) quadrant, T2 would appear equally often in the upper and lower right (left) quadrants. T1 always occurred 1.333 s into the 2.666 s RSVP sequence (the 41st of 80 frames). T2 lagged 400 ms (onset to onset; 12 frames at 33.33 ms/frame) behind T1 on synchronized trials. Asynchronized trials *individually* required T2 lags other than 400 ms, given the quadrant alternation across successive frames. However, the T2 lags *averaged* to 400 ms *across* asynchronized trials. Specifically, on asynchronized trials with ipsilateral targets, T2 lagged randomly either 333 or 467 ms behind T1 (10 or 14 frames at 33.33 ms/frame). On asynchronized trials with contralateral targets, T2 lagged randomly either 367 or 433 ms behind T1 (11 or 13 frames at 33.33 ms/frame).

All other aspects of the stimuli, task, procedure, and data analyses in Experiment 2 matched those in Experiment 1.

## Results

**Fig. 6:**
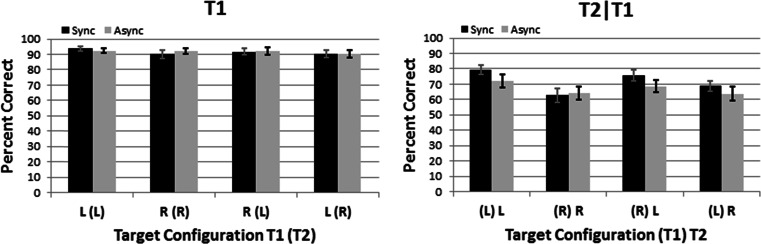
Data from four-stream synchronized versus asynchronized displays. Left and right panelsindicate the percentage correct for T1-identification and T2-identification given a correct T1 response (T2|T1), respectively. Error bars reflect +1 *SEM* (*N* = 26)

Figure 6’s left panel shows virtually identical T1-identification accuracy for synchronous (black columns) and asynchronous (gray columns) displays across target configurations. ANOVAs confirmed that all effects shown in Figure 6’s left panel were nonsignificant. Additionally, T1-idenfication accuracy approached ceiling level in all conditions. These data demonstrate that attention can select salient targets at 15 Hz per hemifield if targets occur at distinct positions within hemifields. Even more notably, the data reveal near-ceiling T1-identification accuracy even though *new* retinal information occurred globally at 30 Hz (i.e., four times the sampling rates observed in some behavioral studies). How might this arise? One possibility is that temporally independent neural events underlie attentional selection of distinct positions within each hemifield. Crucially, these events’ temporal independence would allow them to operate additively. The present data suggest that this additive activity allows attention to operate at 7.5 Hz per location, 15 Hz within a hemifield, and 30 Hz globally, without losing accuracy.

Figure 6’s right panel shows statistically reliable (but modest) main effects of our temporal and spatial manipulations on T2|T1-identification. Regarding our Timing manipulation, although synchronized displays generated significantly greater T2|T1-identification than did asynchronized displays, *F*(1, 25) = 13.7, *p* = .015, _p_η^2^ = 0.35, the difference in percentage correct was modest: 71.7% versus 67.2% correct. We find the modesty of this decrease remarkable, given that we *quadrupled* the asynchronized global new information rate (30 Hz) relative to that in synhcronized displays. Indeed, this 4.5 percentage point cost for quadrupling the global new information rate amounted to just half the cost associated with our T2-side manipulation. That is, repositioning T2 from the LVF (74.0% correct) to the RVF (64.9% correct) reduced T2|T1-identification by 9.1 percentage points. An ANOVA on the main effect of T2-side determined this LVF advantage to be marginally signicant after Bonferroni correction for 15 statistical comparisons, *F*(1, 25) = 9.3, *p* = .075, _p_η^2^ = 0.27.

Overall, Experiment 2 revealed that quadrupling the global *new* information rate from 7.5 to 30 Hz generated little or no accuracy costs in identifying targets that were spatially distributed across visual quadrants. This finding matches what one would expect if visual attention’s neural mechanisms monitored distinct spatial locations in a temporally independent (rather than a phase locked) manner.

## Metamer Control Experiment

In Experiment 2, T1-identification remained virtually identical across synchronized displays and displays asynchronized at 30 Hz. Additionally, when we replaced synchronized displays with displays asynchronized at 30 Hz, mean T2|T1-identification decreased only modestly – from 71.7% to 67.2% correct. What explains such small effect sizes, given that 30 Hz flicker exceeds the presumed 7.5 Hz sampling rate by a factor of four?

One explanation comes from the fact that, in principle, temporally independent neural events could operate additively to achieve higher collective sampling rates. An alternative explanation pertains to the phenomenon of metamers: physically different stimuli that remain perceptually indistinguishable (Wandell, 1995; Williams, Tweten, & Sekuler 1991). In Experiment 2, perhaps the 30 Hz flicker was sufficiently fast to render the asynchronized displays perceptually indistinguishable from (metameric with) the synchronized displays. After all, even our synchronized displays flickered physically (though not perceptually), given the 60 Hz refresh rate of the computer monitor.

Because metamers constitute a failure of discrimination, the metamer hypothesis for Experiment 2’s small effect sizes predicts that participants would fail to discriminate synchronized displays from 30 Hz asynchronized displays (i.e., *d*’ = 0). We tested this prediction in a metamer control experiment.

## Method

### Stimuli

Stimuli in the metamer control experiment comprised the same four-stream synchronized and asynchronized (30 Hz) RSVP sequences shown in Experiment 2. Additionally, from those 80-frame RSVP sequences, we extracted half the frames (frames 21–60) to create a parallel set of 40-frame RSVP sequences presented at half speed (15 Hz rather than 30 Hz). In this way, the entire stimulus set comprised 2.667 s four-stream RSVP displays, either synchronized or asynchronized across quadrants, and presented at either 15 or 30 Hz.

### Task

After viewing each RSVP sequence, participants pressed either the “S” key or the “D” key, respectively, to indicate whether stimuli in the four quadrants flashed at the *same time* or at *different times*. Immediate error feedback followed each response. Participants responded on a standard computer keyboard with no restrictions on which finger or hand to use.

### Procedure

After several practice trials, each participant completed 160 trials for analysis. These trials comprised 40 randomized presentations from each condition in our 2 × 2 design: Timing (synchronized vs. asynchronized displays) by Presentation-Rate (15 vs. 30 Hz displays).

### Data Analysis

For each participant, we used standard procedures from Signal Detection Theory (Green & Swets, 1966) to compute discriminability (*d*’), separately for the 15 and 30 Hz conditions. Operationally, “hits” and “false alarms” occurred when participants made “Different” (D-key) responses to asynchronized and synchronized displays, respectively. A within-subjects *t* test compared *d*’ values in the 15 Hz condition to those in the 30 Hz condition. A one-sample *t* test compared the empirically observed *d*’ values in the 30 Hz condition to the value predicted by the metamer hypothesis: *d*’ = 0.

## Results

**Fig. 7:**
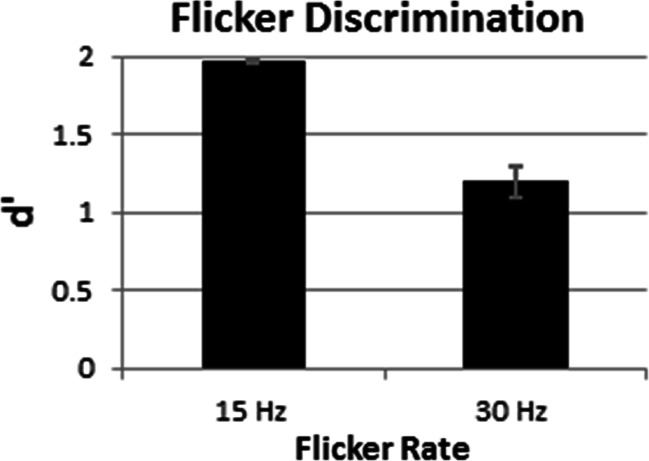
Data from the metamer control experiment. Error bars reflect +1 *SEM* (*N* = 16)

Figure 7 displays three important results from the metamer control experiment. First, *d*’ approached ceiling level when participants discriminated 15 Hz synchronized displays from those with quadrants alternately flickering at 15 Hz. Second, when the flicker rate increased to 30 Hz, flicker discriminability (*d*’) decreased significantly – indeed, by nearly 40%, *t*(15) = 7.5, *p* < .001, _p_η^2^ = 0.79. This decreased discriminability adheres to predictions from the metamer hypothesis. Third, even in the 30 Hz condition, flicker discriminability (*d*’) remained more than 11 standard errors greater than chance. In fact, contrary to the metamer hypothesis, the observed 1.197 *d*’ value in the 30 Hz condition significantly exceeded chance, one-sample *t* test with test value *d*’ = 0, *t*(15) = 11.7, *p* < .001, _p_η^2^ = 0.90, and corresponded to 88% correct without response bias (Green & Swets, 1966).

To summarize, participants in our metamer control experiment reliably discriminated synchronized displays from 30 Hz asynchronized displays (88% correct and more than 11 standard errors greater than chance). This excellent discriminability argues against metamers as an explanation for why Experiment 2’s 30 Hz asynchronized displays generated little or no impairment relative to the 7.5 Hz synchronized displays. Dissimilarly, one would expect such small effect sizes in Experiment 2 if visual attention’s neural mechanisms monitored distinct spatial locations in a temporally independent manner, additively reaching 30 Hz precision across spatially distinct locations.

## Discussion

In the present study, we investigated whether visual attention exhibits phase locking or temporal independence across distinct spatial locations. To distinguish between these possibilities, we required participants to identify two targets (T1 and T2) embedded in multistream RSVPs displayed concurrently across lateral hemifields (Experiment 1) or quadrants (Experiment 2). By experimentally manipulating whether the streams flashed synchronously or asynchronously, we controlled the rate at which new retinal information occurred *globally* while holding the local rate constant. If visual attention operated in a phase-locked manner across distinct spatial locations (i.e., globally), one would expect worse target identification for asynchronous than for synchronous streams. This is because, relative to the synchronous streams, new retinal information in the asynchronous streams occurred two (hemifield case) or four (quadrant case) times faster overall. The asynchronous condition’s globally hastened new information rate would not impair target identification, however, if visual attention operated with temporal independence across distinct spatial locations.

Our primary findings can be summarized as follows. Doubling the global rate of new information from 7.5 to 15 Hz imposed no cost on T1-identification nor on T2|T1-identification (Experiment 1). Quadrupling the global rate of new information from 7.5 to 30 Hz imposed no cost on T1-identification and only modestly impaired T2|T1-identification: 71.7% versus 67.2% correct (Experiment 2). These null and small effects of quadrupling the global presentation rate to 30 Hz occurred even though participants reliably discriminated asynchronous displays flickering at 30 Hz from synchronous nonflickering displays (Metamer Control Experiment). Collectively, these data disconfirm global and hemifield phase locking, each of which predicts worse target identification for asynchronized streams than for synchronized streams. To our knowledge, these experiments are the first to directly test whether visual attention operates in a phase-locked manner across distinct spatial locations.

Our finding that target identification remained largely robust to doubled (Experiment 1) and quadrupled (Experiment 2) global information rates supports temporally independent visual attention across distinct spatial locations. Indeed, these behavioral data align well with cortically focal MEG activity observed when the left and right hemispheres independently entrain to contralateral rhythmic visual stimulation (Spaak et al., 2014). The present behavioral results similarly converge with recent EEG evidence that presenting two targets in rapid succession at distinct locations generates two separate attentional foci, “each with its own *independent* time course” (Eimer & Grubert, 2014, p. 193, emphasis added). Our results extend this finding from two to four foci. Specifically, the fact that target identification remained robust when our global rate of new information quadrupled to 30 Hz points to separate attentional foci across the four quadrants, each with its own independent time course. Notably, physiological studies in owl monkeys (Allman & Kaas, 1974) and humans (DeYoe et al., 1996) reveal that cortical areas V2 and V3 exhibit an organization based on visual quadrants. Consistent with this quadrant-based cortical organization, an earlier behavioral experiment demonstrated a “quandrantic effect” when participants attentively tracked two moving targets: better *inter*-quadrant than *intra*-quadrant performance, while controlling for intertarget distance (Carlson et al., 2007). The cortical organization of areas V2 and V3 therefore plausibly imposes a quadrant-based limit on attentional sampling. As our RSVP results suggest, attentional sampling can operate in a temporally independent manner across quadrants to additively achieve greater global than local sampling rates.

Our RSVP results also revealed a hemifield asymmetry. Specifically, T2|T1-identification exhibited a significant LVF advantage. Repositioning T2 from the RVF to the LVF improved T2|T1-identication by 11.9 and 9.1 percentage points in Experiments 1 and 2, respectively. For comparison, the change in T2|T1-identification generated by switching hemifields (11.9 and 9.1 percentage points) amounted to approximately twice the change (4.5 percentage points) generated by quadrupling the global presentation rate. In other words, the hemifield in which targets appeared mattered more than the global information rate.

The significant LVF advantage observed here likely reflects an asymmetry in visual attention rather than an asymmetry in early stimulus-driven vision. We state this for two reasons. First, we are unaware of any physiological evidence for lateral hemifield asymmetries in the temporal properties of early visual pathway neurons. Second, numerous behavioral studies have shown LVF advantages on tasks that challenge visual attention’s temporal limits (Bosworth et al., 2012; Holländer et al., 2005; Kelly & Matthews, 2011; Matthews et al., 2012; Matthews et al., 2013; Matthews & Welch, 2015; Scalf et al., 2007; Śmigasiewicz et al., 2010; Verleger et al., 2009; Verleger et al., 2010; Verleger et al., 2011; Verleger et al., 2013), and these LVF behavioral advantages have been linked to N2pc attentional markers (Verleger et al., 2011; Verleger et al., 2013). In any case, the significant LVF advantage observed in the present study provides further evidence for a right parietal “when” pathway (Battelli et al., 2007; Battelli et al., 2008; Davis et al., 2009).

We end with an admittedly speculative proposal linking LVF attentional advantages to the previously proposed microconsciousness theory (Zeki & Bartels 1999). Moutoussis described microconsciousness theory as positing that “conscious visual perception is not single and unified but rather made out of several, independent consciousnesses of the different visual attributes” (Moutoussis, 2012, p. 2). Microconsciousness theory aims to explain intriguing psychophysically measured mismatches among attended features. Examples include mismatches between the timing of color and motion changes (Moutoussis & Zeki, 1997) as well as transitivity violations in stereoscopic depth (Farell & Ng, 2014).^3^ Because distinct stimulus attributes such as color, motion, binocular disparity, orientation (and arguably spatial position; Patzwahl & Treue, 2009) register in distinct cortical ensembles, mismatches in feature binding can arise. While the cortically distributed nature of visual feature detectors may generate small but measurable perceptual mismatches under appropriate experimental conditions, it also sets the stage for improving attention’s temporal resolution globally. That is, the temporal independence observed here and the perceptual mismatches that motivated microconsciousness theory might be opposite sides of the same coin. Spatially and temporally independent feature detectors don’t always synchronize perfectly (generating perceptual mismatches) but can additively hasten attention’s global temporal resolution. This may have been an advantageous evolutionary trade-off, rendering the whole faster than its parts.

## Footnotes

1 The supplementary information also contains the percentage of correct T2 identifications, independent of T1 responses. Because these T2-only data exhibited patterns virtually identical to those for T2|T1, we omitted them from the main text for brevity.

2 Informally, readers with music training might recognize the temporal arrangement of our asynchronized trials as “quarter notes” per quadrant, “8th notes” per hemifield, and “16th notes” globally. Correspondingly, the temporal arrangement of our synchronized trials would be “quarter notes” at each of those three spatial scales.

3 A transitivity violation in stereoscopic depth occurs when participants perceive X nearer than Y, Y nearer then Z, but perceive X as either farther than or in the same depth plane as Z (Farell & Ng, 2014).

## References

[CR1] Akyürek EG, Eshuis SA, Nieuwenstein MR, Saija JD, Baskent D, Hommel B (2012). Temporal target integration underlies performance at lag 1 in the attentional blink. Journal of Experimental Psychology: Human Perception and Performance.

[CR2] Akyürek EG, Riddell PM, Toffanin P, Hommel B (2007). Adaptive control of event integration: Evidence from event-related potentials. Psychophysiology.

[CR3] Akyürek EG, Toffanin P, Hommel B (2008). Adaptive control of event integration. Journal of Experimental Psychology: Human Perception and Performance.

[CR4] Allman JM, Kaas JH (1974). The organization of the second visual area (V II) in the owl monkey: A second order transformation of the visual hemifield. Brain Research.

[CR5] Alvarez GA, Cavanagh P (2005). Independent resources for attentional tracking in the left and right visual hemifields. Psychological Science.

[CR6] Alvarez GA, Gill J, Cavanagh P (2012). Anatomical constraints on attention: Hemifield independence is a signature of multifocal spatial selection. Journal of Vision.

[CR7] Anderson DE, Ester EF, Klee D, Vogel EK, Awh E (2014). Electrophysiological evidence for failures of item individuation in crowded visual displays. Journal of Cognitive Neuroscience.

[CR8] Asanowicz D, Smigasiewicz K, Verleger R (2013). Differences between visual hemifields in identifying rapidly presented target stimuli: Letters and digits, faces, and shapes. Frontiers in Psychology.

[CR9] Awh E, Pashler H (2000). Evidence for split attentional foci. Journal of Experimental Psychology: Human Perception and Performance.

[CR10] Battelli L, Cavanagh P, Intriligator J, Tramo MJ, Henaff MA, Michel F, Barton JJ (2001). Unilateral right parietal damage leads to bilateral deficit for high-level motion. Neuron.

[CR11] Battelli L, Cavanagh P, Martini P, Barton JJ (2003). Bilateral deficits of transient visual attention in right parietal patients. Brain.

[CR12] Battelli L, Pascual-Leone A, Cavanagh P (2007). The ‘when’ pathway of the right parietal lobe. Trends in Cognitive Sciences.

[CR13] Battelli L, Walsh V, Pascual-Leone A, Cavanagh P (2008). The ‘when’ parietal pathway explored by lesion studies. Current Opinion in Neurobiology.

[CR14] Bay M, Wyble B (2014). The benefit of attention is not diminished when distributed over two simultaneous cues. Attention, Perception, & Psychophysics.

[CR15] Bosworth RG, Petrich JAF, Dobkins KR (2012). Effects of spatial attention on motion discrimination are greater in the left than right visual field. Vision Research.

[CR16] Busch NA, Dubois J, VanRullen R (2009). The phase of ongoing EEG oscillations predicts visual perception. Journal of Neuroscience.

[CR17] Busch NA, VanRullen R (2010). Spontaneous EEG oscillations reveal periodic sampling of visual attention. Proceedings of the National Academy of Sciences of the United States of America.

[CR18] Capotosto P, Spadone S, Tosoni A, Sestieri C, Romani GL, Della Penna S, Corbetta M (2015). Dynamics of EEG rhythms support distinct visual selection mechanisms in parietal cortex: A simultaneous transcranial magnetic stimulation and EEG study. Journal of Neuroscience.

[CR19] Carlson TA, Alvarez GA, Cavanagh P (2007). Quadrantic deficit reveals anatomical constraints on selection. Proceedings of the National Academy of Sciences of the United States of America.

[CR20] Chakravarthi R, Cavanagh P (2009). Bilateral field advantage in visual crowding. Vision Research.

[CR21] Chakravarthi R, Vanrullen R (2012). Conscious updating is a rhythmic process. Proceedings of the National Academy of Sciences of the United States of America.

[CR22] Chun MM (1997). Temporal binding errors are redistributed by the attentional blink. Perception & Psychophysics.

[CR23] Chun MM, Potter MC (1995). A two-stage model for multiple target detection in rapid serial visual presentation. Journal of Experimental Psychology: Human Perception and Performance.

[CR24] Davis B, Christie J, Rorden C (2009). Temporal order judgments activate temporal parietal junction. Journal of Neuroscience.

[CR25] DeYoe, E. A., Carman, G. J., Bandettini, P., Glickman, S., Wieser, J., Cox, R.,… Neitz, J. (1996). Mapping striate and extrastriate visual areas in human cerebral cortex. *Proceedings of the National Academy of Sciences of the United States of America, 93*(6), 2382–2386. 10.1073/pnas.93.6.2382 PMC39805 8637882

[CR26] Dubois J, Hamker FH, VanRullen R (2009). Attentional selection of noncontiguous locations: The spotlight is only transiently “split”. Journal of Vision.

[CR27] Dugué, L., & Vanrullen, R. (2014). The dynamics of attentional sampling during visual search revealed by Fourier analysis of periodic noise interference. *J Vis, 14*(2). doi:10.1167/14.2.11 10.1167/14.2.11 24525262

[CR28] Eimer M, Grubert A (2014). Spatial attention can be allocated rapidly and in parallel to new visual objects. Current Biology.

[CR29] Farell B, Ng C (2014). Perceived depth in non-transitive stereo displays. Vision Research.

[CR30] Forster B, Corballis PM, Corballis MC (2000). Effect of luminance on successiveness discrimination in the absence of the corpus callosum. Neuropsychologia.

[CR31] Goodbourn PT, Holcombe AO (2015). “Pseudoextinction”: Asymmetries in simultaneous attentional selection. Journal of Experimental Psychology: Human Perception and Performance.

[CR32] Gray MJ, Frey HP, Wilson TJ, Foxe JJ (2015). Oscillatory recruitment of bilateral visual cortex during spatial attention to competing rhythmic inputs. Journal of Neuroscience.

[CR33] Green, D. M., & Swets, J. W. (1966). Signal detection theory and psychophysics. New York: John Wiley & Sons

[CR34] Hanslmayr S, Aslan A, Staudigl T, Klimesch W, Herrmann CS, Bauml KH (2007). Prestimulus oscillations predict visual perception performance between and within subjects. NeuroImage.

[CR35] Hanslmayr S, Volberg G, Wimber M, Dalal SS, Greenlee MW (2013). Prestimulus oscillatory phase at 7 Hz gates cortical information flow and visual perception. Current Biology.

[CR36] Holländer A, Corballis MC, Hamm JP (2005). Visual-field asymmetry in dual-stream RSVP. Neuropsychologia.

[CR37] Hopf JM, Luck SJ, Girelli M, Hagner T, Mangun GR, Scheich H, Heinze HJ (2000). Neural sources of focused attention in visual search. Cerebral Cortex.

[CR38] Isaak MI, Shapiro KL, Martin J (1999). The attentional blink reflects retrieval competition among multiple rapid serial visual presentation items: Tests of an interference model. Journal of Experimental Psychology: Human Perception and Performance.

[CR39] Jans B, Peters JC, De Weerd P (2010). Visual spatial attention to multiple locations at once: The jury is still out. Psychological Review.

[CR40] Jefferies LN, Enns JT, Di Lollo V (2014). The flexible focus: whether spatial attention is unitary or divided depends on observer goals. Journal of Experimental Psychology: Human Perception and Performance.

[CR41] Kawahara J, Kumada T, Di Lollo V (2006). The attentional blink is governed by a temporary loss of control. Psychonomic Bulletin and Review.

[CR42] Kelly, J. G., & Matthews, N. (2011). Attentional oblique effect when judging simultaneity. *Journal of Vision, 11*(6), 1–15. doi:10.1167/11.6.10 10.1167/11.6.10 21602558

[CR43] Landau AN, Fries P (2012). Attention samples stimuli rhythmically. Current Biology.

[CR44] Marshall TR, O’Shea J, Jensen O, Bergmann TO (2015). Frontal eye fields control attentional modulation of alpha and gamma oscillations in contralateral occipitoparietal cortex. Journal of Neuroscience.

[CR45] Mathewson KE, Gratton G, Fabiani M, Beck DM, Ro T (2009). To see or not to see: Prestimulus alpha phase predicts visual awareness. Journal of Neuroscience.

[CR46] Matthews, N., Vawter, M., & Kelly, J. G. (2012). Right hemifield deficits in judging simultaneity: a perceptual learning study. *Journal of Vision, 12*(2), 1–14. doi:10.1167/12.2.1 10.1167/12.2.1 22303023

[CR47] Matthews, N., Welch, L., Festa, E., & Clement, A. (2013). Remapping time across space. *Journal of Vision, 13*(8), 1–15. doi:10.1167/13.8.2 10.1167/13.8.2 23818678

[CR48] Matthews, N., Welch., L., (2015). Left visual field attentional advantage in judging simultaneity and temporal order. *Journal of Vision, 15*(2), 1–13. doi:10.1167/15.2.7 10.1167/15.2.7 25761346

[CR49] McMains SA, Somers DC (2004). Multiple spotlights of attentional selection in human visual cortex. Neuron.

[CR50] McMains SA, Somers DC (2005). Processing efficiency of divided spatial attention mechanisms in human visual cortex. Journal of Neuroscience.

[CR51] Mishkin M, Ungerleider LG (1982). Contribution of striate inputs to the visuospatial functions of parieto-preoccipital cortex in monkeys. Behavioural Brain Research.

[CR52] Moutoussis K (2012). Asynchrony in visual consciousness and the possible involvement of attention. Frontiers in Psychology.

[CR53] Moutoussis K, Zeki S (1997). A direct demonstration of perceptual asynchrony in vision. Proceedings of the Royal Society B: Biological Sciences.

[CR54] Niebur E, Koch C, Rosin C (1993). An oscillation-based model for the neuronal basis of attention. Vision Research.

[CR55] Olivers CN, Hilkenmeier F, Scharlau I (2011). Prior entry explains order reversals in the attentional blink. Attention, Perception, & Psychophysics.

[CR56] Olivers CN, Van der Stigchel S, Hulleman J (2007). Spreading the sparing: Against a limited-capacity account of the attentional blink. Psychological Research.

[CR57] Patzwahl DR, Treue S (2009). Combining spatial and feature-based attention within the receptive field of MT neurons. Vision Research.

[CR58] Reardon KM, Kelly JG, Matthews N (2009). Bilateral attentional advantage on elementary visual tasks. Vision Research.

[CR59] Reeves A, Sperling G (1986). Attention gating in short-term visual memory. Psychological Review.

[CR60] Rorden C, Mattingley JB, Karnath HO, Driver J (1997). Visual extinction and prior entry: Impaired perception of temporal order with intact motion perception after unilateral parietal damage. Neuropsychologia.

[CR61] Scalf PE, Banich MT, Kramer AF, Narechania K, Simon CD (2007). Double take: Parallel processing by the cerebral hemispheres reduces attentional blink. Journal of Experimental Psychology: Human Perception and Performance.

[CR62] Śmigasiewicz K, Shalgi S, Hsieh S, Moller F, Jaffe S, Chang CC, Verleger R (2010). Left visual-field advantage in the dual-stream RSVP task and reading-direction: A study in three nations. Neuropsychologia.

[CR63] Spaak E, De Lange FP, Jensen O (2014). Local entrainment of alpha oscillations by visual stimuli causes cyclic modulation of perception. Journal of Neuroscience.

[CR64] Spalek TM, Falcon LJ, Di Lollo V (2006). Attentional blink and attentional capture: Endogenous versus exogenous control over paying attention to two important events in close succession. Perception and Psychophysics.

[CR65] Spalek TM, Lagroix HE, Yanko MR, Di Lollo V (2012). Perception of temporal order is impaired during the time course of the attentional blink. Journal of Experimental Psychology: Human Perception and Performance.

[CR66] Tichener EB (1910). A textbook of psychology.

[CR67] VanRullen R, Carlson T, Cavanagh P (2007). The blinking spotlight of attention. Proceedings of the National Academy of Sciences of the United States of America.

[CR68] VanRullen R, Dubois J (2011). The psychophysics of brain rhythms. Frontiers in Psychology.

[CR69] VanRullen R, Koch C (2003). Is perception discrete or continuous?. Trends in Cognitive Science.

[CR70] Verleger R, Dittmer M, Śmigasiewicz K (2013). Cooperation or competition of the two hemispheres in processing characters presented at vertical midline. PLoS ONE.

[CR71] Verleger R, Möller F, Kuniecki M, Śmigasiewicz K, Groppa S, Siebner HR (2010). The left visual-field advantage in rapid visual presentation is amplified rather than reduced by posterior-parietal rTMS. Experimental Brain Research.

[CR72] Verleger R, Smigasiewicz K, Moller F (2011). Mechanisms underlying the left visual-field advantage in the dual stream RSVP task: evidence from N2pc, P3, and distractor-evoked VEPs. Psychophysiology.

[CR73] Verleger R, Sprenger A, Gebauer S, Fritzmannova M, Friedrich M, Kraft S, Jaśkowski P (2009). On why left events are the right ones: Neural mechanisms underlying the left-hemifield advantage in rapid serial visual presentation. Journal of Cognitive Neuroscience.

[CR74] Visser TA (2015). Expectancy-based modulations of lag-1 sparing and extended sparing during the attentional blink. Journal of Experimental Psychology: Human Perception and Performance.

[CR75] Wandell BA (1995). Foundations of vision.

[CR76] Wever E (1949). Theory of hearing.

[CR77] Wever E, Bray C (1937). The perception of low tones and the resonance-volley theory. The Journal of Psychology: Interdisciplinary and Applied.

[CR78] Williams D, Tweten S, Sekuler R (1991). Using metamers to explore motion perception. Vision Research.

[CR79] Womelsdorf, T., & Fries, P. (2007). The role of neuronal synchronization in selective attention. *Curr Opin Neurobiol, 17*(2), 154–160. doi:10.1016/j.conb.2007.02.002 10.1016/j.conb.2007.02.002 17306527

[CR80] Woo SH, Kim KH, Lee KM (2009). The role of the right posterior parietal cortex in temporal order judgment. Brain and Cognition.

[CR81] Wyble B, Bowman H, Nieuwenstein M (2009). The attentional blink provides episodic distinctiveness: Sparing at a cost. Journal of Experimental Psychology: Human Perception and Performance.

[CR82] Wyble B, Potter MC, Bowman H, Nieuwenstein M (2011). Attentional episodes in visual perception. Journal of Experimental Psychology: General.

[CR83] Zeki S, Bartels A (1999). Toward a theory of visual consciousness. Consciousness and Cognition.

